# Targeting mitophagy in diabetic retinopathy: novel insights into SQSTM1/BNIP3L pathway regulated by luteolin

**DOI:** 10.3389/fphar.2025.1593213

**Published:** 2025-06-25

**Authors:** Shuyan Zhang, Jiajun Wu, Yinjian Zhang

**Affiliations:** Department of Ophthalmology, Longhua Hospital, Shanghai University of Traditional Chinese Medicine, Shanghai, China

**Keywords:** diabetic retinopathy, monocytes, Sqstm1, Bnip3L, luteolin

## Abstract

**Objective:**

Diabetic retinopathy (DR) is a leading microvascular complication of diabetes. Luteolin, a flavonoid with known anti-inflammatory and antioxidant properties, has demonstrated therapeutic potential in early investigations for the treatment of DR. However, its precise molecular mechanisms remain inadequately defined. This study aimed to explore the local and systemic immunological mechanisms underlying luteolin’s therapeutic effects on DR.

**Methods:**

Key regulatory genes and cell subpopulations were identified from single-cell RNA sequencing (scRNA-Seq) datasets derived from peripheral blood mononuclear cells (PBMCs) and retinal tissues of DR patients. The molecular interactions were analyzed using molecular docking simulations. Reactive oxygen species (ROS) were quantified through DCFDA assays, while retinal structural damage was assessed using Hematoxylin and eosin (H&E) and Periodic Acid-Schiff (PAS) staining. Comprehensive analyses, including enzyme-linked immunosorbent assays (ELISA), immunofluorescence, immunohistochemistry, and Western blotting were conducted to evaluate cytokine levels and protein expression.

**Results:**

The study revealed that luteolin exerted protective effects against DR primarily by activating mitophagy and reducing oxidative stress, with the SQSTM1/BNIP3L pathway emerging as a critical mediator. Furthermore, a novel mechanistic link was established between monocyte activity and DR progression, highlighting the VISFATIN signaling pathway’s role in immune cell regulation and its contribution to disease pathology.

**Conclusion:**

This study offers novel insights into the luteolin’s therapeutic potential in DR, particularly activating mitophagy through the SQSTM1/BNIP3L axis, which expands the scope of natural compounds in addressing this sight-threatening complication of diabetes.

## 1 Introduction

Diabetic retinopathy (DR) is usually insidious and asymptomatic in the early stages, but it might rapidly develop into a sight-threatening condition or even blindness (Antonetti et al., 2021). Common clinical features of DR include microaneurysms, retinal hemorrhages and pathologic retinal neovascularization Dulull et al. (2019), which has led to a greater focus on various vascular cell types, particularly endothelial cells (ECs) and pericytes. However, DR is not only an ocular disease, but is also associated with systemic inflammation and immune response. Cell subsets in the peripheral blood may serve as mirrors reflecting the patient’s systemic immune status. Furthermore, sustained hyperglycemic state leads to increased reactive oxygen species (ROS) production and mitochondrial dysfunction. Autophagy is a catabolic process induced by oxidative stress, which is essential for maintaining cell stability and survival under stress (Dehdashtian et al., 2018). One study conducted on db/db mice revealed that Panax ginseng saponin R1 mitigated DR’s pathological alterations through PINK1-mediated mitochondrial autophagy (Zhou et al., 2019). Furthermore, in retinal pigment epithelial cells induced by high glucose levels, melatonin was observed to downregulate the expression of PINK, BNIP3 and NIX to preserve mitochondrial homeostasis in DR (Zhang et al., 2019).

In contrast to laser photocoagulation and vitreoretinal surgery, pharmacological treatment has emerged as a viable alternative treatment for DR. Calcium dobesilate is the only antioxidant for oral administration in clinical therapy (Liu et al., 2019), while anti-VEGF agents remain the primary drug for intravitreal injections (Flaxel et al., 2020). However, the expensive costs and long-term treatment involved with synthetic pharmaceuticals have prompted renewed interest in the exploration of natural plant-derived compounds, which typically present a more favorable safety profile with reduced toxicity and fewer adverse effects (fister et al., 2011; Li et al., 2025; Yan et al., 2023). Numerous investigations have underscored the remarkable restorative potential of natural plant extracts in ameliorating blood-retinal barrier (BRB) injury (Criollo-Mendoza et al., 2023; Ren et al., 2024). Luteolin is a natural flavonoid derived from various botanical plants, which has been shown to possess significant anti-inflammatory, antioxidant, anti-apoptotic and neuroprotective activities (Zhu et al., 2024; He et al., 2023; Huang et al., 2023). Preliminary studies have disclosed that luteolin may exert protective effects on retinal cells by modulating inflammatory pathways, oxidative stress and blood lipids and blood sugar status (Li et al., 2019; Yang et al., 2021; Zhang et al., 2024). However, the precise molecular mechanism between luteolin, mitochondrial autophagy and DR remains to be elucidated. Our study will endeavor to uncover the comprehensive therapeutic effects of luteolin on diverse DR pathology aspects. The study design includes a combination of single-cell RNA sequencing (scRNA-Seq) computational analysis, along with *in vitro* experimental validation.

**FIGURE 1:**
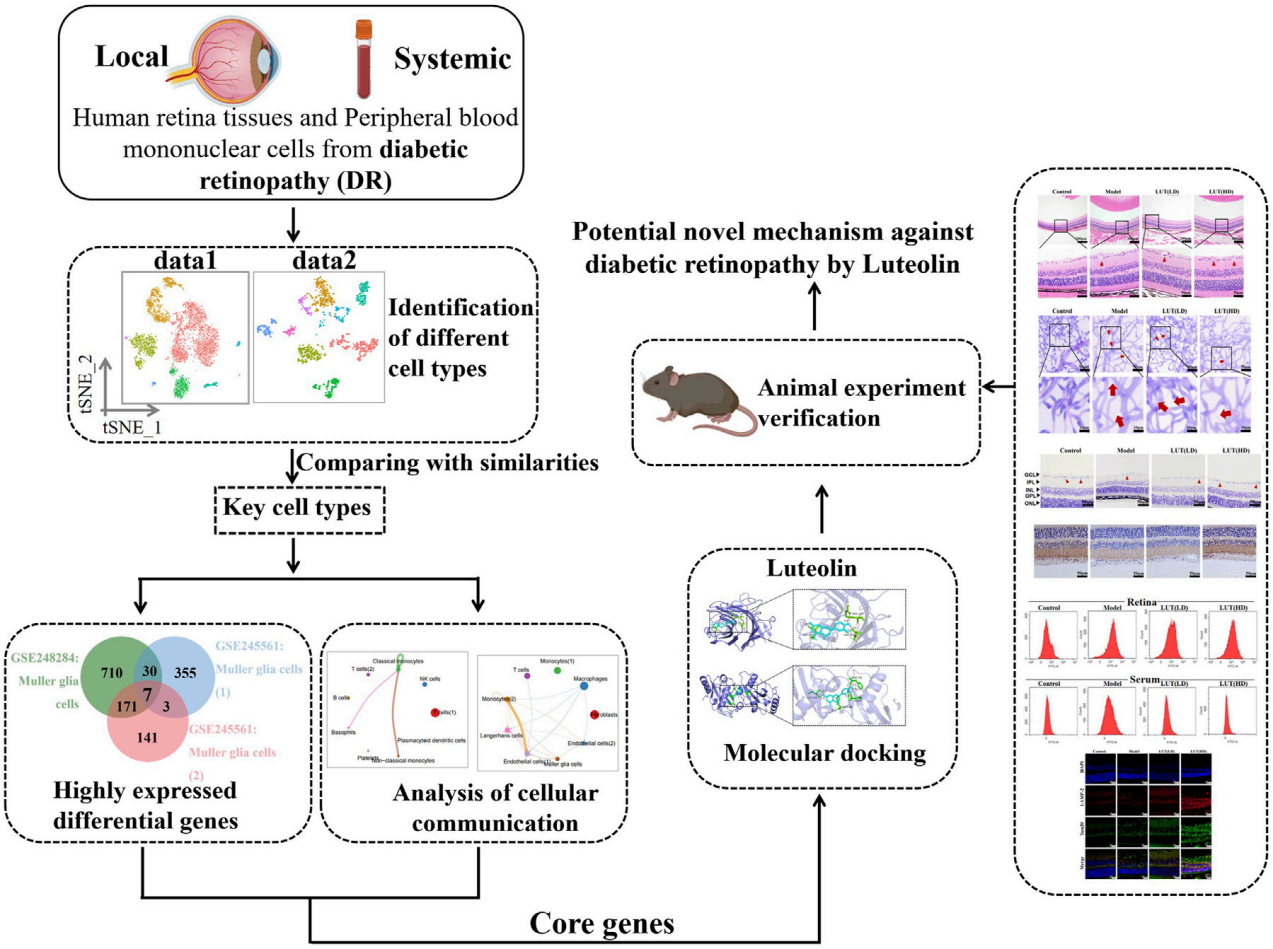
Flow chart of the study. Key regulatory genes and cell subpopulations were identified from scRNA-Seq datasets. Then, molecular docking and *in vivo* experiment was conducted to verify the findings.

A critical aspect of our research is a comparison of scRNA-Seq results from peripheral blood and retinal tissues, which revealed the intricate interplay between systemic immune responses and local tissue damage in DR (Figure 1). It is a breakthrough that we elucidated the novel involvement of the SQSTM1/BNIP3L pathway in the molecular pathogenesis of DR, which was traditionally related to cancers, cardiovascular diseases and autoimmune conditions (Li et al., 2021; Poon et al., 2021). In our study, the SQSTM1/BNIP3L pathway emerged as a pivotal regulator in critical cellular processes, particularly oxidative stress and mitophagy within the context of DR. These findings underscored the delicate equilibrium in cellular functions, expanding current knowledge and opening avenues for targeted therapeutic interventions in the management of DR.

## 2 Materials and methods

### 2.1 Data acquisition and quality control

The scRNA-seq datasets (Homo Spanies) were downloaded from the Gene Expression Omnibus (GEO) database, including GSE248284 (Peripheral blood mononuclear cells (PBMCs) from DR patients), GSE245561 (fibrovascular membrane from DR patients). Using the R package (version 4.3.2) Seurat (version 4.4.0), the GSE248284 were filtered with the quality control (nCount_RNA<10,000, 500<nFeature_RNA<2000, percent. mitochondrial<10%), while GSE245561 (nCount_RNA< 15,000, 500<nFeature_RNA < 5,000, percent. mitochondrial<10%) were filtered for subsequent analyse.

### 2.2 Normalization, clustering and annotation

All samples were individually normalized with the “NormalizeData” function and genes with significant expression variability were selected to perform Principal Component Analysis (PCA) to reduce dimensionality. The cell clusters were annotated based on Cell Markers database in conjunction with previously reported markers (Corano et al., 2023; Liao et al., 2024). Additionally, we applied unified parameters to analyse the two datasets, to ensure consistency and comparability across two datasets.

### 2.3 Functional enrichment analysis of DEGs

Gene ontology (GO) and Kyoto Encyclopedia of Genes and Genomes (KEGG) pathway enrichment analysis were performed to reveal uncharted biological function among key cell clusters. The R package “clusterProfiler” (version 4.10.0) could be applied to conduct functional enrichment analysis on genes with the most significantly adjusted p-value and fold change.

### 2.4 Molecular docking

The 3D structures of BNIP3L (PDB code: 4GKY) and SQSTM1 (PDB code: 2ZJD) were retrieved from the PDB database (https://www.rcsb.org), and water molecules were removed by Pymol 2.5.5. Luteolin (CID: 5280445) structures were used for ligand docking and downloaded from the PubChem compound database. Hydrogenation was conducted by AutoDockTools1.5.6, with the active pocket position duly configured.

### 2.5 Induction of DR in mice and treatment

The experiment was ethically approved by the Institution Animal Ethics Committee of Longhua Hospital Shanghai University of Traditional Chinese Medicine (FM-IA-008). 8-week-old male C57/BL6 mice (22–23 g) were purchased from Chengdu Dashuo Experimental Animal Co., Ltd. China (SCXY 2020-034). Animal studies followed the ARRIVE 2.0 guidelines. Following continuous intraperitoneal administration of STZ solution (55 mg/kg) for 5 days, blood glucose levels were monitored for a week. Mice with blood glucose levels higher than 15 mmol/L for three consecutive occasions were recognized as diabetic mice in the study.

Subsequently, untreated non-diabetic mice (n = 6) were categorized as the normal control. Diabetic mice were randomly divided into three groups. Untreated diabetic mice (n = 6) were considered as the model group. Afterwards, the remaining mice with hyperglycemia were administered two doses of Luteolin (99.51%, HY-N0162, MCE, United States): 25 mg/kg (n = 6) and 50 mg/kg (n = 6) via daily gavage for 8 weeks. The control and model groups received daily ddH_2_O for 8 weeks. At the end of the study, the mice were anaesthetized using isoflurane delivered through a vaporizer set to 4% for induction. Euthanasia was performed upon confirmation of deep anesthesia, characterized by the absence of pedal reflex, utilizing an overdose of pentobarbital (200 mg/kg) via intraperitoneal injection to ensure humane euthanasia.

### 2.6 Measurement of weight, RBG and IPGTT

To evaluate the hypoglycemic effects of luteolin, we utilized a glucometer (Bayer, Germany) to measure random blood glucose (RBG) levels and conducted an intraperitoneal glucose tolerance test (IPGTT). An electronic scale (maximum range: 200 g, accuracy: 0.01 g) was employed to record the weight. Weight and RBG assessments were performed every 2 weeks while blood glucose levels remained above 15 mmol/L. IPGTT was measured after injecting blood glucose (2 g/kg) at 0.5, 1, 1.5 and 2 h. The glucose tolerance capacity of the mice was evaluated by computing the area under the curve (AUC) and plotting it based on the relevant time points in each group.

### 2.7 Measurement of retinal vascular permeability

Evans Blue (EB) dye (E2129, Sigma, United States, 45 mg/kg) was administered intravenously via the tail vein of each mouse. A cannula was inserted into the left ventricle after 120 min circulation. Each mouse was injected with 4% paraformaldehyde at 66 mL/min for 2 min to remove the dye. Then, the eye was removed and the retina is separated. EB was extracted by incubating each retina in 0.12 mL formaide for 18 h at 70°C. The extracts were centrifuged at 4°C at 70,000 g for 45 min. Absorbance of the filtrate was measured using a microplate reader (SpectraMAX Plus384, Molecular Devices, United States) at 620 nm and 720 nm.

### 2.8 Hematoxylin and eosin (H&E) staining

Paraffin-embedded eyeballs were dewaxed with toluene, dehydration and staining with hematoxylin. After washing, the slices were separated with 1% hydrochloric acid alcohol, dyed with eosin and washed with distilled water for 10 min. The prepared tissue sections were examined under a digital trinocular camera microscope (BA210Digital, Motic, China) for detailed observation and analysis.

### 2.9 Periodic acid-schiff (PAS) staining

The paraffin-embedded eyeballs were immersed in 4% paraformaldehyde solution for 72 h, followed by submersion in PBS (PH = 7.4) overnight. The retinas were peeled off and digested with 3% trypsin solution at 37°C for 2 h. Then, the isolated retinal microvascular network was affixed to a slide for PAS staining.

### 2.10 Immunohistochemistry (IHC) staining

After a series of deparaffinization and rehydration steps on paraffin-embedded eyeballs, the tissues were incubated with the primary polyclonal antibody anti-ZO-1 (GB111981, Servicebio, China, 1:100) at 37°C overnight. Subsequently, HRP-labeled goat anti-rabbit IgG (GB23303, Servicebio, China, 1:100) was incubated for 60 min.

### 2.11 Immunofluorescence (IF) staining

After dewaxed and hydrated procedures, paraffin-embedded eyeballs were incubated in QuickBlock™ Blocking Buffer (P0260, Beyotime, China) for 30 min at room temperature. Then, the anti-TOM20 (11802-1-AP, Proteintech, United States, 1:100), anti-LAMP-2 (bs-2379R, Bioss, China, 1:100), or LC3B (14600-1-AP, Proteintech, United States, 1:100) were incubated at 4°C overnight. Finally, the sections were incubated for 1–2 h with HRP-labeled goat anti-rabbit IgG (GB23303, Servicebio, China, 1:100) in the dark. Immunofluorescence images were observed under a fluorescence microscope OlyVIA (Olympus, Tokyo, Japan).

### 2.12 Measurement of ROS level

According to the manufacturer’s procedures, reactive oxygen species (ROS) production levels in retinal tissues and serum were analyzed by staining with 2′, 7′ -dichlorofluorescein-diacetate (DCFH-DA, Beyotime, China).

### 2.13 Biochemical detection

Enzyme-linked immunosorbent assay (ELISA) was used to calculate the anti-oxidative stress values of luteolin *in vivo* by the corresponding kit: MDA (A003-1-2), SOD (A001-3-1), GSH-Px (A005-1-2, Nanjing Jiancheng Institute of Bioengineering, China).

### 2.14 Western blot analysis

The RIPA kit (P0013B, Beyotime, China) was used to extract all proteins from retinal tissues and protein quantification was calculated by the BCA kit (P0009, Beyotime, China). Followed with electrophoresis, membrane transfer (PVDF, Sigma, United States), and incubated with the appropriate amount of primary antibody at 4°C overnight: anti-BNIP3L (DF8163, Affinity, United States, 1:3,000), anti-COX IV (A6564, Abclonal, China, 1:5,000), anti- SQSTM1 (A19700, Abclonal, China, 1:1,000), anti-Beclin1 (A7353, Abclonal, China, 1:2,000), anti-β-actin (AC026, Abclonal, China, 1:50,000). Then, the goat anti-rabbit IgG (H + L) HRP (S0001, Affbiotech, China, 1:5,000) was incubated at room temperature for 2h. Finally, the ECL color development solution (k22030; Abbkine, United States) was utilized for chromogenic detection.

### 2.15 Statistical analysis

Statistical analysis was performed through GraphPad Prism version 8.0 software. All results were presented as the mean ± SD. *P < 0.05, **P < 0.01, and ***P < 0.001 were considered statistically significant. One-way analysis of variance (ANOVA) followed by Tukey’s *post hoc* test was used for multiple group comparisons. Adjusted *p*-values (<0.05) were considered statistically significant.

## 3 Results

### 3.1 Characterization of cell types in diabetic retinopathy PBMCs and retinas

**FIGURE 2:**
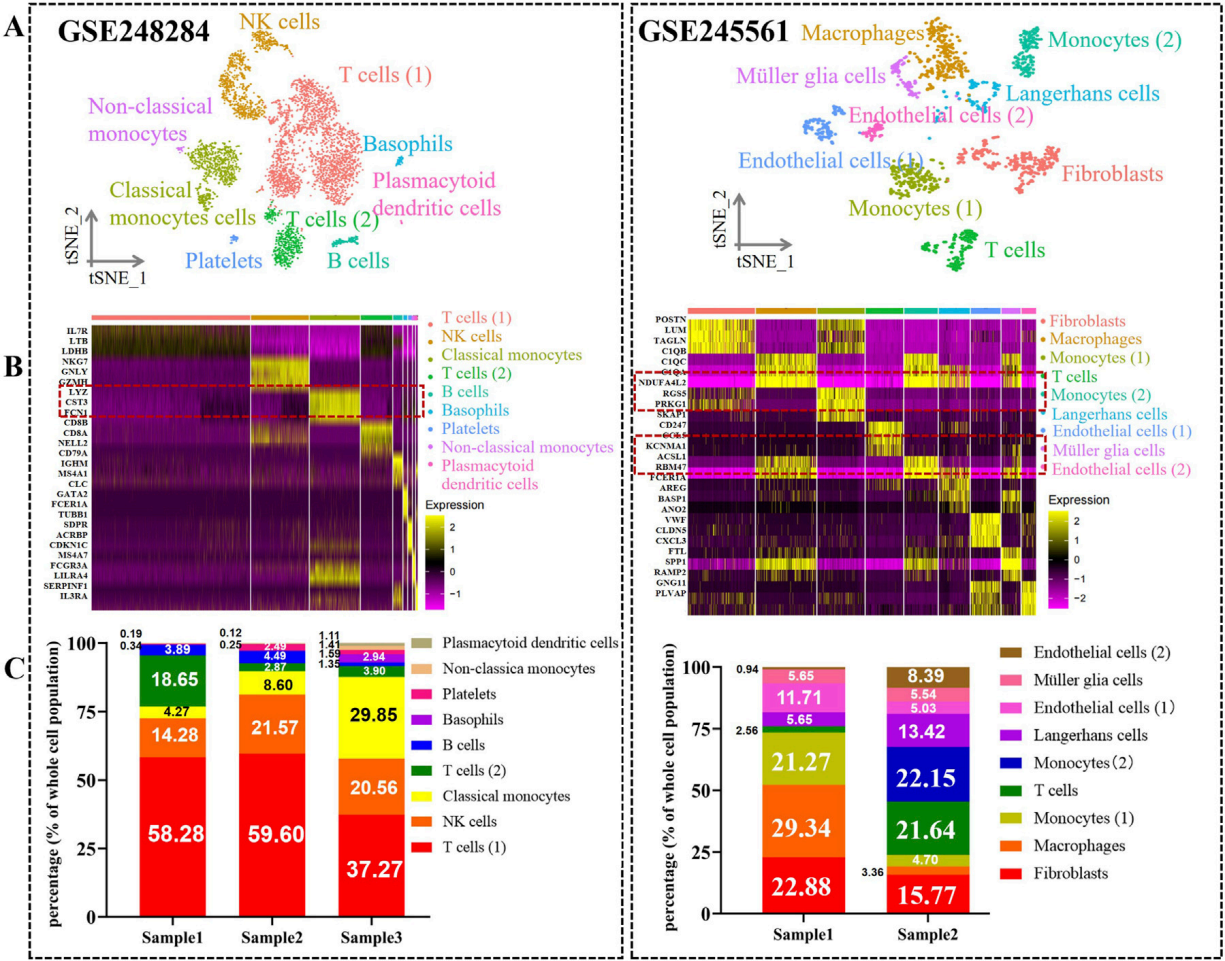
Atlas of cell types from DR in PBCs and retina tissues. **(A)** TSNE plots show nine cell various clusters in GSE248284 and GSE245561, respectively. **(B)** Heatmap of top three marker genes in each cluster among two datasets. The marker genes in monocytes are highlighted. **(C)** Bar plots demonstrate the distribution of cell type proportions in GSE248284 and GSE245561.

Based on rigorous quality control and cell filtering, we severally identified 18,246 and 22,222 features for further comprehensive biological analysis. Following normalization and dimensionality reduction procedures, we performed t-distributed stochastic neighbor embedding (t-SNE) to exhibit the nine distinct clusters, separately (Figure 2A, Supplementary Table S1). Upon comparison with the two datasets, monocytes and T cells were the common clusters. As shown in Figure 2B, a heatmap was constructed to delineate the key genes in each cell cluster. Monocytes were characterized by LYZ, CST3, CTSS, NDUFA4L2, KCNMA1, and ACSL1. T cells exhibited high expression levels in IL7R, LTB, CD8A, CD52, CCL5, and CD69. The distribution of cell type proportions revealed that the highest percentage of monocytes was up to 29.85% and 22.15% (Figure 2C, Supplementary Table S2), while monocytes were unevenly distributed across the samples in DR patients. Meanwhile, mounting evidences exhibited the pivotal role of monocytes in contributing to chronic inflammation and neovascularization in the pathological process of DR. Thus, we specifically chose to focus on monocytes for a deeper analysis.

### 3.2 Biological features of monocytes in patients with DR

**FIGURE 3:**
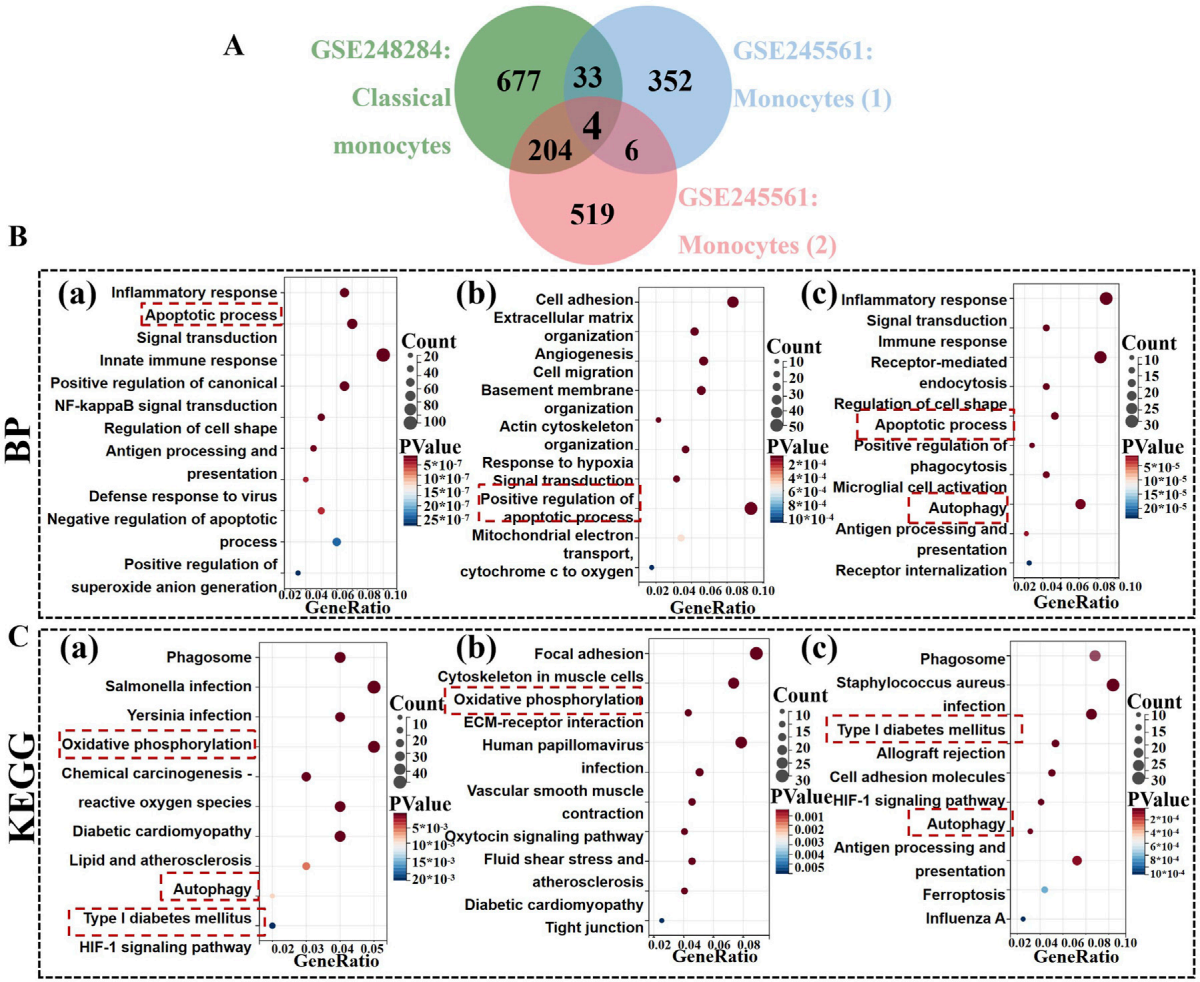
Identification of highly expressed gene in monocytes among two datasets. **(A)** The Venn diagrams show four common genes (SQSTM1, BNIP3L, FOS, MEF2C) in monocytes between GSE248284 and GSE245561. **(B)** GO analysis of highly expressed genes in monocytes. **(C)** KEGG analysis of highly expressed genes in monocytes. **(a)** monocytes in the GSE24828; **(b,c)** two monocyte types in the GSE245561.

To elucidate the biological characteristics of monocytes in PBCs and retinal tissues pertinent to DR, we performed differentially expressed genes (DEGs) analyses between the two datasets. As shown in Figure 3A, 918 genes were significantly upregulated in PBC samples. In addition, 395 and 733 genes were severally identified as highly expressed in DR retina tissues (Supplementary Table S3). Subsequently, functional enrichment analyses were employed to further investigate the roles and regulatory mechanisms of these DEGs within monocytes. With regard to the GO analyses conducted on two datasets, monocytes were significantly enriched in “0006915”: Apoptotic process (*P* = 5.74*10^−15^, *P* = 1.44*10^−7^), “0043065: positive regulation of apoptotic process” (*P* = 4.88*10^−5^); “0006954”: inflammatory response (*P* = 4.93*10^−15^, *P* = 2.69*10^−11^) (Figure 3B). Moreover, monocytes were closely associated with “0006123: Mitochondrial electron transport (*P* = 1.14*10^−4^)” and “0006914: Autophagy (*P* = 0.003)” in DR retina samples (Supplementary Table S4). These findings indicated that monocytes contribute to both systemic and local pathological processes in DR by apoptosis, inflammation, mitochondrial function and autophagy-related processes. Additionally, KEGG analysis demonstrated that monocytes were strongly relevant to: “hsa00190: Oxidative phosphorylation (*P* = 2.32*10^−9^, *P* = 0.007)”; “hsa04140: Autophagy (*P* = 5.12*10^−4^, *P* = 0.001)”; “hsa04940: Type I diabetes mellitus (*P* = 8.88*10^−4^, *P* = 4.20*10^−7^)” (Figure 3C, Supplementary Table S5). The results indicated that monocytes may play critical roles in oxidative stress, autophagic pathways and immune processes, offering insights into potential therapeutic targets aimed at mitigating monocyte-driven inflammation and cellular damage in DR.

### 3.3 Deciphering the complexity of the VISFATIN signaling pathway in monocytes

**FIGURE 4:**
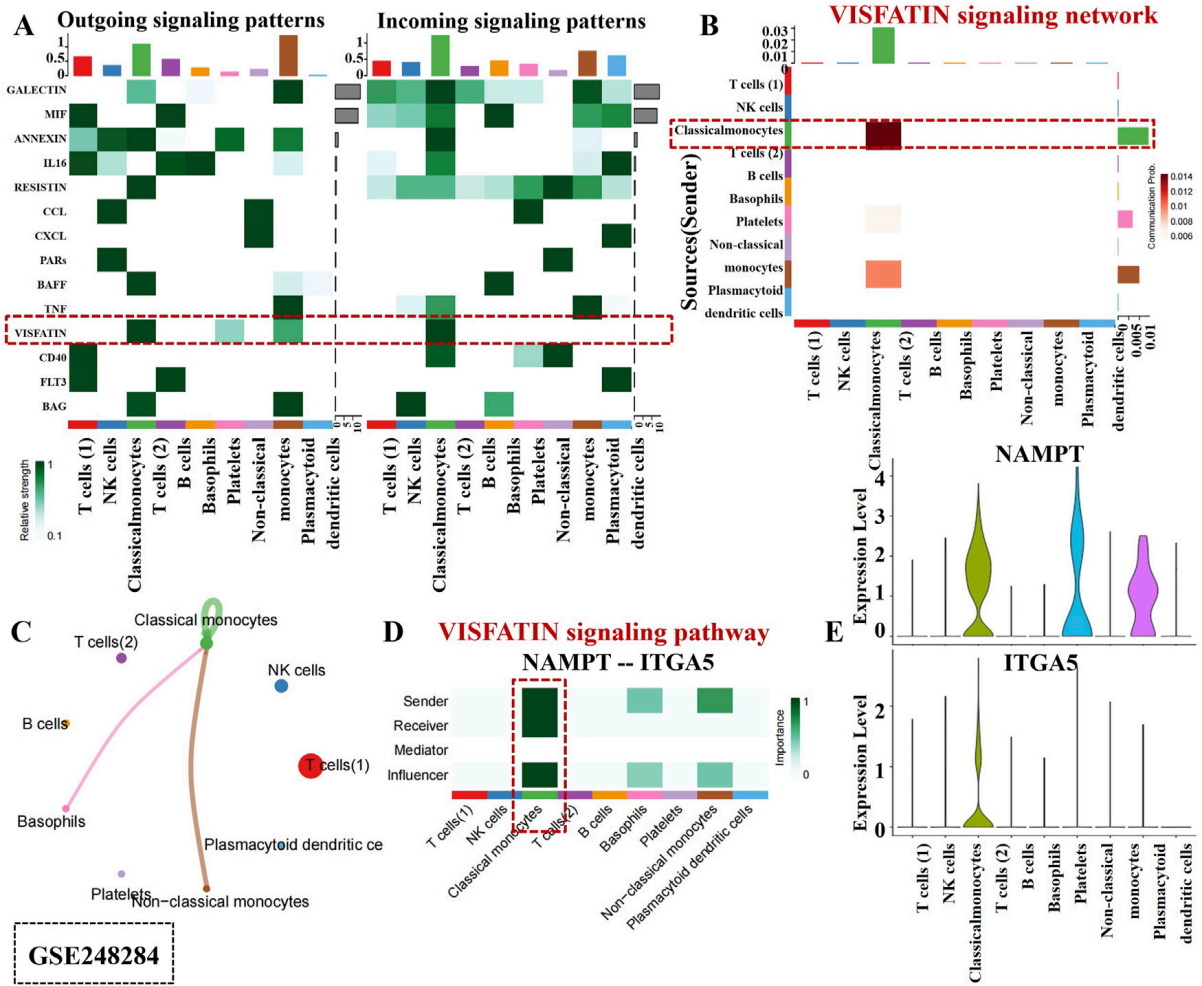
Cell-cell communication inferred by VISFATIN signaling pathway in PBCs. **(A)** Outgoing and incoming signal strength of each signaling pathway in each cell population in GSE248284. **(B)** Heatmap of the VISFATIN signaling pathway–mediated intercellular communication intensity. **(C)** Circle plot demonstrated intercellular communication network associated with the VISFATIN signaling pathway. The thickness of the line and color represent the communication signal strength. **(D)** Heatmap showed the sender, receiver, mediator and influencer of VISFATIN signaling pathway network. **(E)** Expression level of the VISFATIN signaling ligand and receptor (NAMPT - ITGA5) in each cell population.

Intercellular communication networks are essential for coordinating cellular functions through various signaling interactions. Our analysis of scRNA-seq data from PBMC samples revealed that monocyte owned numerous strong self-regulating functions within the VISFATIN signaling pathway, compared to the other 14 signaling pathways (Figure 4A). As shown in Figure 4B, only monocytes received VISFATIN signaling inputs, indicating a direct and specific influence of VISFATIN signaling on the monocyte cellular landscape. Furthermore, our analysis illustrated that autocrine signaling predominantly mediated intercellular interactions within the VISFATIN pathway, while basophils and non-classical monocytes clusters exhibited significant paracrine activity (Figure 4C). Centrality analysis of the VISFATIN signaling network (NAMPT - ITGA5) revealed that monocytes primarily acted as signal senders, exerting influence on themselves through autocrine loops (Figure 4D). In terms of ligand-receptor gene expression, both NAMPT and ITGA5 were highly expressed in monocytes, exhibiting a crucial function in modulating their activities and signaling processes (Figure 4E).

**FIGURE 5:**
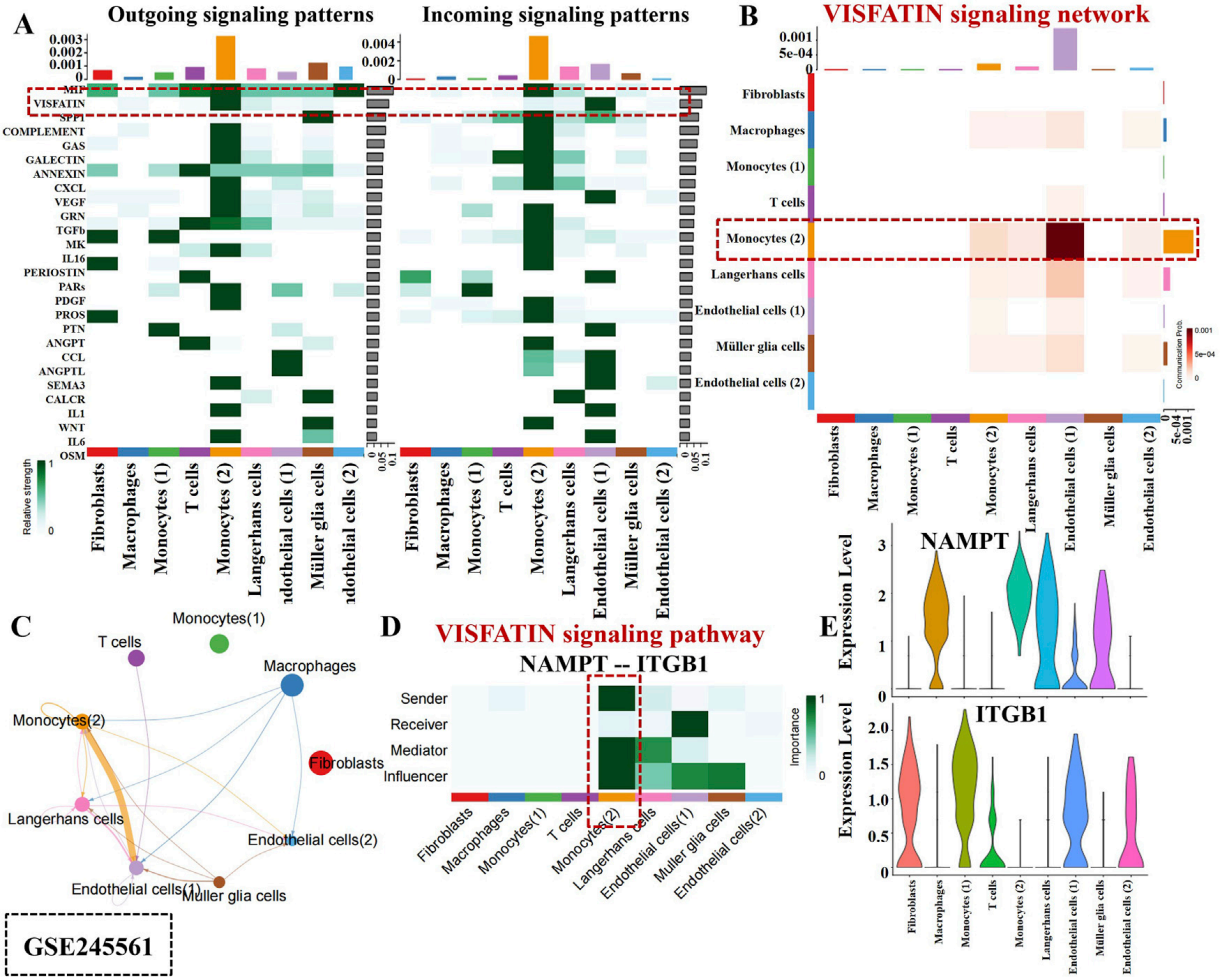
Intercellular communication mediated by VISFATIN signaling pathway in retina tissues. **(A)** Outgoing and incoming signal strength of each signaling pathway in each cell population in GSE245561. The line thickness and color exhibited the communication signal strength. **(B)** A heatmap showed the VISFATIN signaling pathway–mediated intercellular communication intensity. **(C)** Circle plot illustrates intercellular communication network associated with the VISFATIN signaling pathway. **(D)** Heatmap displayed the sender, receiver, mediator and influencer of VISFATIN signaling pathway network. **(E)**. Expression level of the VISFATIN signaling ligand and receptor (NAMPT–ITGB1) in each cell population.

In the Figure 5A, a robust interaction was observed among monocytes within the VISFATIN signaling pathway. Additionally, VISFATIN signaling was identified as the primary source for most cell clusters in the DR retina tissues (Figure 5B). Notably, monocytes directed towards ECs by VISFATIN signaling, which indicated their pivotal role in modulating vascular responses in DR. Figure 5C further elucidated these signaling interactions. Monocytes exhibited momentous paracrine and autocrine signaling capabilities, indicating their dual roles in communicating with neighboring cells and self-regulating their own functions.

Interestingly, monocytes acted as major senders, mediators and influencers within this network, thereby controlling communication and exerting substantial effect on ECs (Figure 5D). Additionally, our analysis uncovered that NAMPT and ITGB1 involved in critical functional processes and signaling pathways in connection with monocyte activity (Figure 5E). Collectively, the above results indicated monocytes potential contributions to the DR occurrence within the VISFATIN signaling pathway. Deciphering these intricate intercellular communications could pave the way for identifying novel therapeutic targets aimed at modulating VISFATIN signaling pathways.

### 3.4 Unveiling highly expressed gene candidates in monocytes across both datasets

**FIGURE 6:**
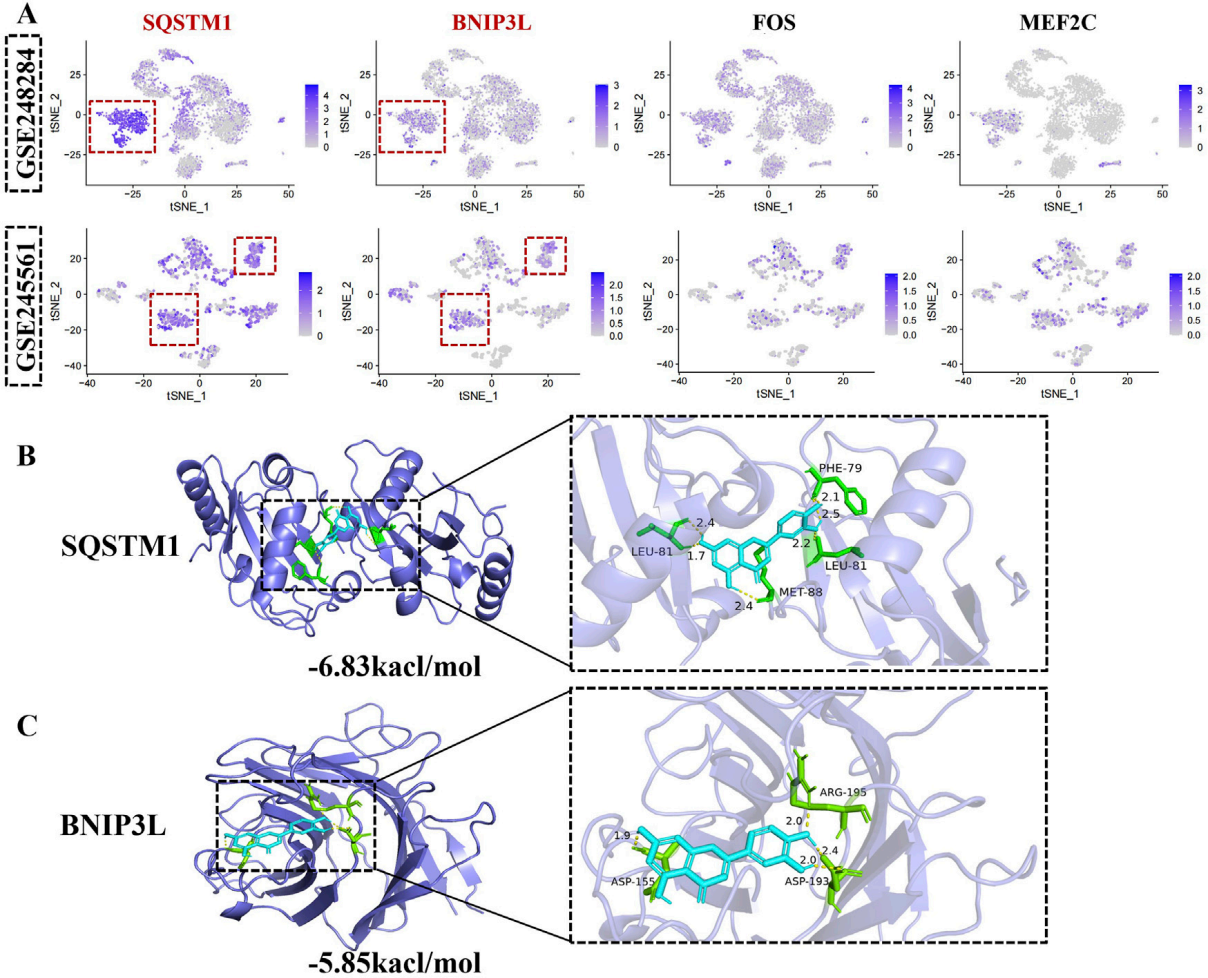
Unveiling hub gene in monocytes across both datasets. **(A)** The t-SNE plots demonstrate the expression of SQSTM1, BNIP3L, FOS, MEF2C in identified cell clusters. The upper panel is GSE248284 and the below panel represents GSE245561. **(B)** Molecular docking of SQSTM1 with luteolin. **(C)** Molecular docking of BNIP3Lwith luteolin. Blue color represents luteolin.

Venn diagrams revealed 4 core genes: SQSTM1, BNIP3L, FOS, MEF2C (Figure 3A). To further explore their expression profiles, we performed t-SNE analyses on both datasets, which demonstrated that SQSTM1 and BNIP3L were more specifically expressed in monocytes (Figure 6A). Consequently, SQSTM1 and BNIP3L emerged as key candidate genes potentially linked to the pathogenesis of DR. Given these genes potential role in DR, we sought to explore whether luteolin, a flavonoid with known anti-inflammatory properties, could directly target SQSTM1 and BNIP3L. However, the precise interaction between luteolin and these genes remained unclear, prompting us to perform molecular docking analysis to gain deeper insight into their possible involvement against DR.

According to the VINA auto-docking software, the binding energies for all ligand-receptors were all less than −5 kcal/mol, indicating spontaneous binding interactions. As shown in Figure 6B, luteolin was found to bind to the amino acid residues LEU-81, MET-88, and PHE-79 of SQSTM1, increasing protein structural stability. Additionally, luteolin interacted with BNIP3L at key binding sites, including ASP-155, ASP-193 and ARG-195, further supporting a strong ligand-receptor interaction (Figure 6C). Luteolin is bound tightly to both SQSTM1 and BNIP3L, highlighting its potential as a therapeutic agent in the modulation of monocyte-related pathways in DR.

### 3.5 High-dose of luteolin exhibited remarkable weight loss reversed and hypoglycemic effect on STZ-induced diabetic mice

**FIGURE 7:**
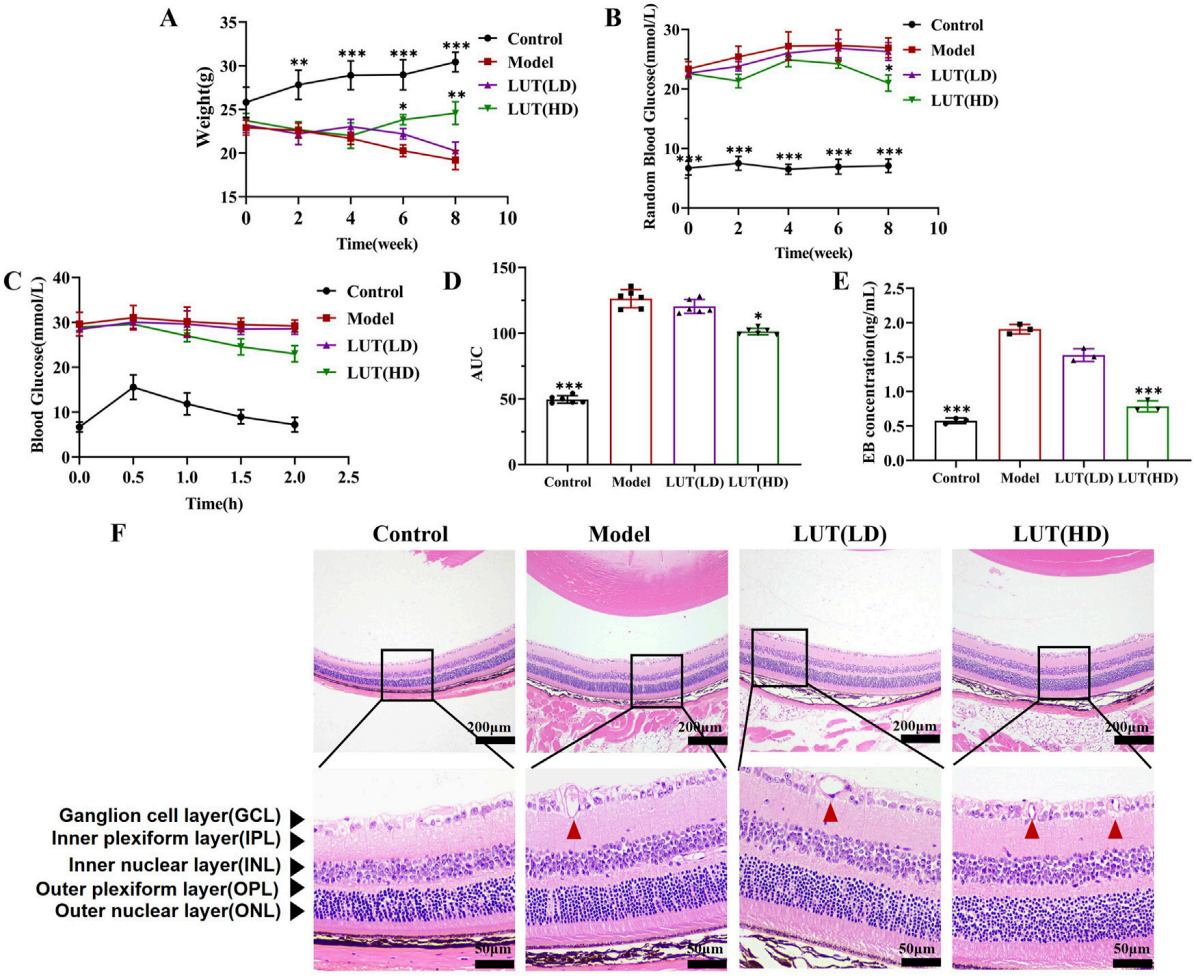
High-dose of luteolin exhibited remarkable weight loss reversal and hypoglycemic effect with protecting retinal structure degeneration in STZ-induced diabetic mice. **(A)** Changes in body weight within various treatments. **(B)** Measurement of random blood glucose. **(C,D)** Intraperitoneal glucose tolerance test and its relevant areas under the curve (AUC) in diabetic mice. **(E)** Detecting retinal vascular permeability through the Evans Blue (EB) leakage in the diabetic mice retina. **(F)** The pathological damage of retinal tissue was observed by H&E staining. Scale: 50 μm and 200 μm. All data are presented as the mean ± SD. LUT (LD): LUT 25 mg/kg; LUT (HD): LUT 50 mg/kg *P < 0.05; **P < 0.01; and ***P < 0.001 versus model.

Initial blood glucose levels and body weight of both STZ-induced diabetic mice and the untreated control groups were observed have no significant differences at baseline (*P* > 0.05, Table 1). As shown in Figure 7A, high-dose luteolin ameliorated weight loss in STZ-induced diabetic mice after 6 weeks of gavage (*P* < 0.05 vs. model). Hyperglycemia is a critical factor in the pathogenesis of DR, which primarily induces oxidative stress and chronic inflammation. In this study, the results of RBG, IPGTT, and AUC demonstrated that high-dose luteolin could exert a significant hypoglycemic effect in the later stages of DR mice (Figures 7B–D, *P* < 0.05 vs. model). Our findings confirmed that high-dose luteolin might induce hypoglycemic activity and ameliorate weight loss for DR treatment.

**TABLE 1:** C57/BL6 mice baseline characteristics.

Group	Weight	Random blood glucose
untreated control mice	22.18 ± 0.74	6.43 ± 0.79
STZ-induced diabetic mice	21.77 ± 0.62^&^	6.73 ± 0.54^&^

^&^ *P >* 0.05 versus untreated control mice.

### 3.6 High-dose of luteolin alleviated retinal layer thickness and loss of RGC in diabetic mice

**FIGURE 8:**
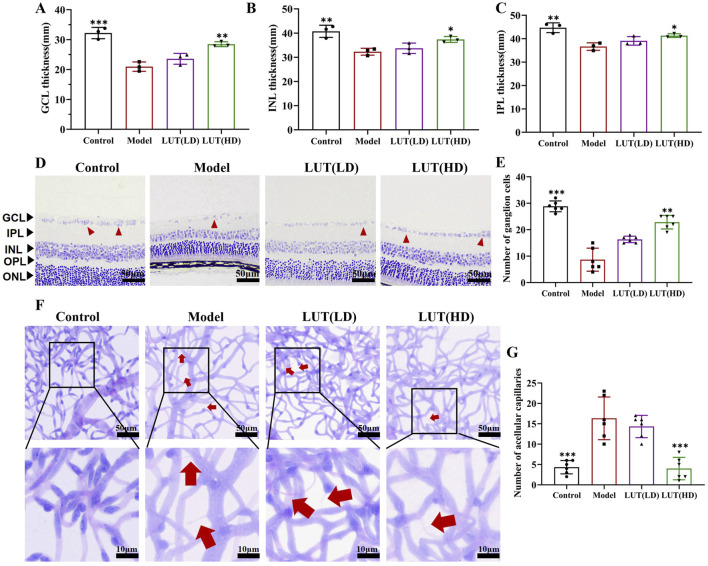
High-dose of luteolin alleviated retinal layer thickness, loss of RGC and microvascular injury in diabetic mice. **(A**–**C)** Measurement of thickness of GCL, INL and IPL with H&E staining respectively. GCL, ganglion cell layer; INL, inner nuclear layer; IPL, inner plexiform layer. **(D,E)** Quantification of Tar violet staining stained RGC cells. Scale: 50 μm. **(F,G)** PAS staining was performed to detect acellular capillaries. Typical performance is amplified. All data are presented as the mean ± SD. LUT (LD): LUT 25 mg/kg; LUT (HD): LUT 50 mg/kg *P < 0.05; **P < 0.01; and ***P < 0.001 versus model.

To further evaluate the protective effects of luteolin on retinal tissue, we examined blood-retinal-barrier permeability. Notably, EB leakage was decreased in the high-dose luteolin intervention group compared to the model group (*P* < 0.001 vs. model, Figure 7E). Previous studies on DR have documented neuronal degeneration in human and mammalian retinas, including reduced thickness of the ganglion cell layer (GCL), inner nuclear layer (INL) and inner plexiform layer (IPL), as well as progressive loss of retinal ganglion cells (RGCs). Consistent with these findings, we measured the thickness of the GCL, INL and IPL in HE-stained retinal sections (Figure 7F). A remarkable reduction in GCL, INL and IPL thickness was observed in untreated diabetic mice, which could be reversed via high-dose luteolin treatment (*P* < 0.05). In contrast, low-dose luteolin had no significant protective effect on the loss of reduced GCL, INL and IPL thickness in STZ-induced diabetic mice (*P* > 0.05). Furthermore, high-dose luteolin improved capillary dilation within the nerve fibre layer of the retina in diabetic mice (Figures 8A–C). Additionally, tar violet staining revealed a substantial loss of RGCs in the model group, which was significantly mitigated by high-dose luteolin treatment (Figures 8D,E, *P* < 0.01 vs. model). These results suggested that high-dose luteolin may exert a neuroprotective effect in DR by preserving retinal layer integrity and preventing RGC loss.

### 3.7 Effective role in ameliorating microvascular injury

**FIGURE 9:**
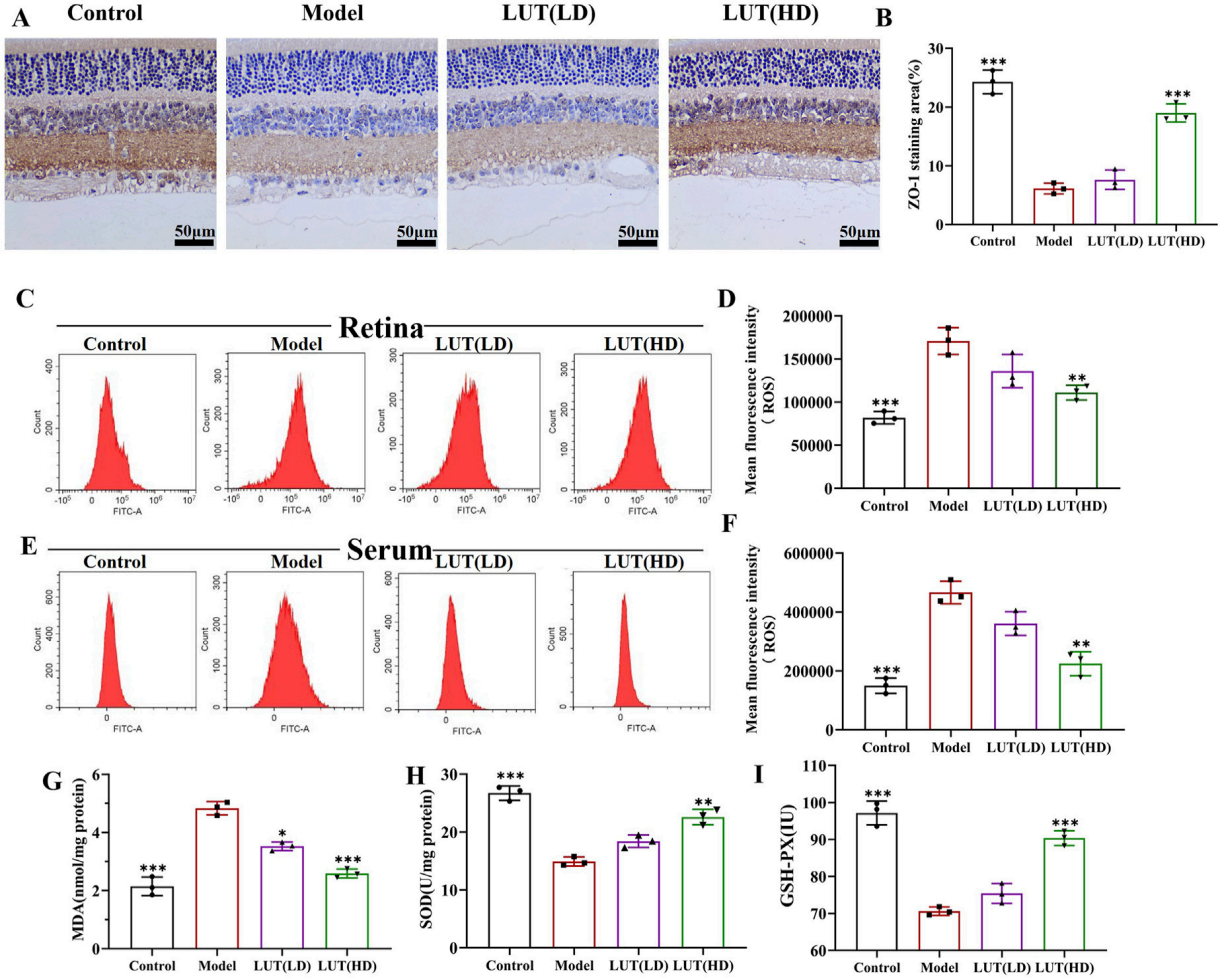
Effective role of luteolin in tight junction and oxidative stress. **(A,B)** ZO-1 expression was measured by immunohistochemistry in retina sections. Scale: 50 μm. **(C,D)** ROS levels in the retina tissues of diabetic mice were measured using flow cytometry. **(E,F)** ROS generation in the serum of diabetic mice was tested by flow cytometry. **(G**–**I)** Detecting the anti-oxidative stress (MDA) and anti-oxidative stress (SOD and GSH-Px) values of luteolin in DR treatment by enzyme-linked immunosorbent assay. All data are presented as the mean ± SD. LUT (LD): LUT 25 mg/kg; LUT (HD): LUT 50 mg/kg *P < 0.05; **P < 0.01; and ***P < 0.001 versus model.

As is widely recognized that DR is a microvascular disease, PAS staining was performed to quantify retinal microvascular impairment. Increased acellular capillaries were observed in the model group, which was a characteristic feature of microvascular injury in DR (Figures 8F,G). Notably, this microvascular impairment was prominently reduced after high-dose luteolin treatment, demonstrating its protective effects on retinal vasculature. Concurrently, IHC was leveraged to assess the potential of luteolin in relieving retinal damage in diabetic mice. In this study, a noteworthy decline was observed in the expression of ZO-1 in the diabetic mice retinas, the difference is significant by high-dose luteolin intervention (Figures 9A,B). While low-dose of luteolin displayed suboptimal efficacy in rectifying the number of acellular capillaries and fortifying tight junctions.

### 3.8 Reduction in ROS production and oxidative stress

Elevated levels of ROS were detected in both retina and serum under high-glucose conditions. However, when diabetic mice were treated with high-dose luteolin, there was an apparent reduction in ROS levels (*P* all <0.05 vs. model) (Figures 9C–F). ROS accumulation triggered oxidative stress, leading to cellular damage and dysfunction. MDA, SOD and GSH-Px were crucial indicators that reflected the potential antioxidant capacity and peroxidative damage. As shown in Figures 9H,I, high-dose luteolin significantly enhanced the activities of SOD and GSH-PX but MDA expressions dramatically declined (all *P* < 0.05 vs. model) (Figure 9G). Integrating with all the results, luteolin may serve as a prospective therapy in DR progression.

### 3.9 Luteolin increased colocalization of Tom20 with LAMP-2 and LC3B in retinae of diabetic mice

**FIGURE 10:**
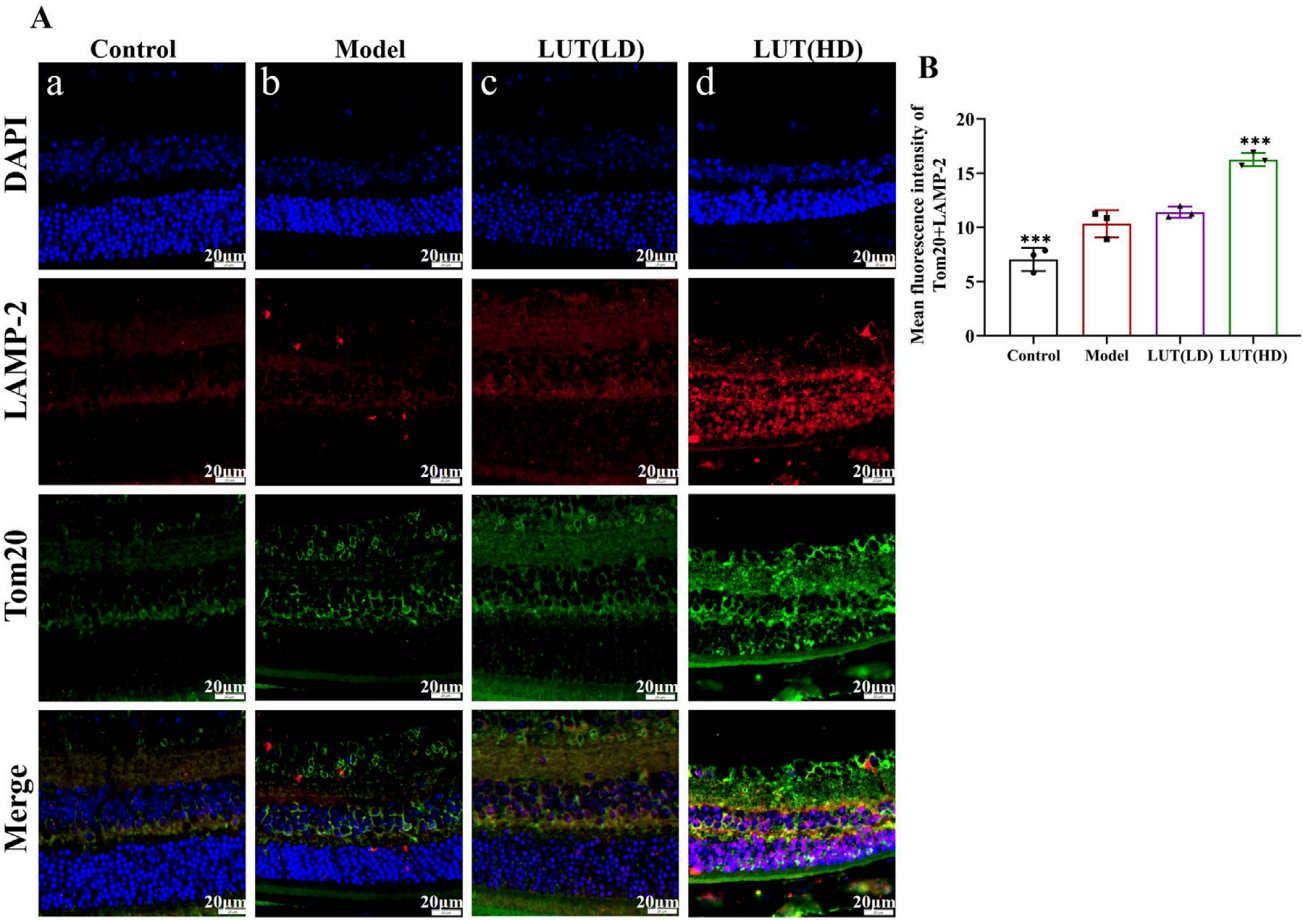
Luteolin promote the upregulation of LAMP-2/Tom20 in STZ-induced diabetic mice. **(A,B)** Immunofluorescence of LAMP-2 and Tom20. Scale: 20 μm. Cells were immunostained with LAMP-2 (red), Tom20 (blue) and DAPI (blue). All data are presented as the mean ± SD. LUT (LD): LUT 25 mg/kg; LUT (HD): LUT 50 mg/kg *P < 0.05; **P < 0.01; and ***P < 0.001 versus model.

To further shed light on the underlying mechanisms of luteolin for retinal damage improvement, confocal laser scanning microscopy was employed to visualize the co-localization of Tom20 with LAMP-2 and LC3B, key markers involved in mitophagy. In untreated diabetic mice, a dramatic increase in LAMP-2/Tom20 and LC3B/Tom20 positive signals was observed in the retina compared to the control group (Figures 10Aa,b, 11Aa,b). This performance clarified the compensatory upregulation of mitophagy in response to mitochondrial damage. Following treatment with high-dose luteolin, a further enhancement in the expression of LAMP-2/Tom20 and LC3B/Tom20 was detected (Figures 10Ad,B, 11Ad,B). These findings indicated that luteolin not only restored mitochondrial function but also actively promoted mitophagy. The elevated co-localization of LAMP-2 with Tom20 demonstrated enhanced lysosomal degradation of damaged mitochondria, while the increased LC3B/Tom20 interaction points to heightened autophagosome formation around defective mitochondria.

**FIGURE 11:**
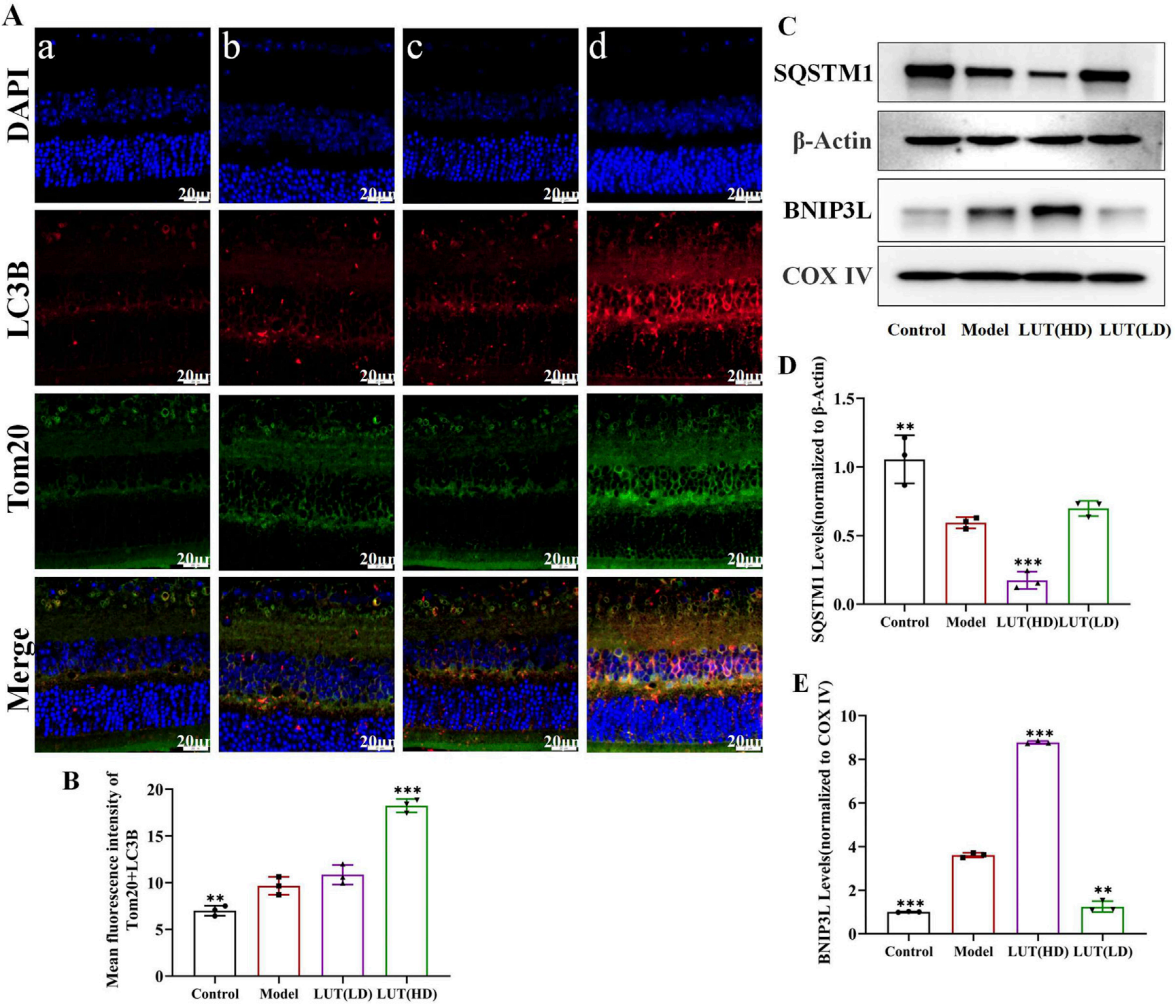
Luteolin promoted mitochondrial autophagy in the retina of diabetic mice by activating SQSTM1/BNIP3L signaling pathway. **(A,B)** Colocalization of LC3B and Tom20 Immunofluorescence. Scale: 20 μm. Cells were immunostained with LAMP-2 (red), Tom20 (blue) and DAPI (blue). **(C)** Expression of SQSTM1, BNIP3L at protein levels in diabetic mice. **(D,E)** Bar charts show the normalized relative to β-Actin and COX IV protein expression. All data are presented as the mean ± SD. LUT (LD): LUT 25 mg/kg; LUT (HD): LUT 50 mg/kg *P < 0.05; **P < 0.01; and ***P < 0.001 versus model.

### 3.10 Effect of luteolin on SQSTM1/BNIP3L pathway activation *in vitro* assays

Western blotting results showed that SQSTM1 expression was reduced in diabetic mice (Figures 11C, D) and further significantly decreased following the high-dose luteolin treatment. SQSTM1 is a crucial autophagy receptor, and its decreased expression may indicate altered mitophagy, contributing to the clearance of damaged mitochondria and oxidative stress reduction. BNIP3L, another critical player in mitophagy, was found to be obviously upregulated under high-glucose conditions, and its expression was further increased after treatment with high-dose luteolin (Figures 11C–E). These results indicated that decreased SQSTM1 and increased BNIP3L were likely involved in the mechanisms through which luteolin exerted its protective effects against DR.

## 4 Discussion

In recent decades, researchers have identified a myriad of compounds derived from natural plant medications with outstanding therapeutic potential for retinal diseases, including curcumin, quercetin, apigenin and resveratrol (Rahman et al., 2021; Wong et al., 2022). Luteolin, an emerging drug, has shown promising preliminary results in ameliorating retinal damage in DR. The intricate pathogenesis of DR involves complex interactions between systemic immune responses and localized tissue injury, necessitating a deeper exploration of undefined mechanisms for effective therapeutic intervention. Recent advancements in scRNA-seq technology have enabled researchers to elucidate the cellular and molecular dynamics underpinning DR, providing detailed insights into the heterogeneity of immune responses in peripheral blood and retinal tissue. However, it remained largely undiscovered whether there exists any overlap or similarity in those findings. In this study, by analyzing GSE248284 and GSE243100, we highlighted monocytes physiological function on peripheral immune activation exacerbating retinal injury in DR. Importantly, the SQSTM1/BNIP3L pathway emerged as a critical mediator of these effects, suggesting that luteolin’s action extends beyond mere symptom alleviation to address underlying molecular mechanisms. This dual perspective expanded our knowledge of the complex interactions that contribute to DR pathology and paved the ways for targeted interventions.

Increasing evidence has shown the pivotal role of circulating immune cells in the DR pathophysiology (Pan et al., 2021; Xu and Chen, 2017). Early-stage DR was characterized by an increase in leukocyte adhesion within the retinal vascular system. Previous studies have emphasized the involvement of T cells in DR pathology, as they secreted pro-inflammatory cytokines that induce ECs damage and apoptosis, thereby exacerbating vascular changes in retinas (Huang and Zhou, 2022). Notably, a cross-sectional study involving 3,277 diabetic patients have demonstrated that decreased levels of peripheral blood monocytes were associated with an increased DR likelihood (Wan et al., 2020). Additionally, a case-control study of 247 patients with type 2 diabetes mellitus (T2DM) indicated that the ratio of monocytes to lymphocytes could serve as a predictive marker for DR (Yue et al., 2015). Longitudinal assessments involving 758 T2DM patients further revealed a correlation between monocyte chemotactic protein-1 levels (MCP-1) and the progression of DR (Ninomiya et al., 2015). These findings suggested that timely monocyte level measurement may aid in the early screening of DR. While previous investigations primarily established clinical associations between DR pathology and monocytes, they might lack direct evidence to infer causal relationships. However, animal experiments have shown that monocyte activation and proliferation contribute to the release of inflammatory mediators (NF-κB, TNF-α, and IL-6), which adversely break microvascular integrity (Aicha et al., 2023; Li and Fan, 2023). Consequently, this study concentrated on the potential of monocytes as a novel therapeutic target for DR management.

A thorough intercellular communication analysis was conducted to disclose the VISFATIN signaling pathway mediating the interactions among monocytes. VISFATIN, a novel adipokine, exerts a crucial function in glucose homeostasis by reducing hepatic glucose release and stimulating glucose utilization in peripheral tissues (Wang et al., 2014). Its expression was notably elevated in the diabetic rat retinas, progressively increasing with disease duration (Qu et al., 2016). Furthermore, under high-glucose conditions, VISFATIN promoted angiogenesis in RF/6A cells by upregulating the VEGF/VEGFR-2 signaling pathway (Chen et al., 2021). Nicotinamide adenine dinucleotide (NAD) serves as an indispensable coenzyme involved in cellular redox reactions, with NAMPT acting as a regulatory factor for intracellular NAD levels. NAMPT participated in several pathological processes by modulating oxidative stress, apoptosis, inflammation, lipid and glucose metabolism (Garten et al., 2015). Relevant research has demonstrated that manipulating the NAMPT-NAD + axis might prevent vascular aging in experimental DR Cappellani et al. (2025). Additionally, recent studies have revealed that the Nampt-Sirt6 axis was critical in extracellular matrix remodeling during diabetic nephropathy (Muraoka et al., 2019). The ITGA5 subunit predominantly formed a heterodimer with ITGB1, culminating in the α5β1 integrin complex, which was crucial for cell adhesion and signaling pathways involved in proliferation, migration, invasion and metastasis (Zhu et al., 2021). An investigation indicated that KAT1 triggered YTHDF2-mediated ITGB1 mRNA instability, thereby mitigating the progression of DR (Qi et al., 2021). While numerous studies about ITGA5 focused on immune infiltration in tumors, it has also been shown that acute loss of ITGA5 in ECs could compromise the retinal barrier integrity (Ayloo et al., 2022). This multifaceted investigation into the VISFATIN signaling pathway and integrin interactions underscored their potential as therapeutic targets in managing DR treatment.

SQSTM1 served as a key regulator for cellular homeostasis, involving various cellular processes, including autophagy, oxidative stress and metabolic reprogramming (Kageyama et al., 2021; Zhang et al., 2018). Abundant research has underscored that deficiencies in SQSTM1 were associated with pathological events linked to mitochondrial dysfunction, emphasizing its critical role in maintaining cellular health (Jakobi et al., 2020; Gao et al., 2020). BNIP3L, a protein localized to the outer mitochondrial membrane, was initially implicated in mediating mitochondrial autophagy during the development of erythroid progenitor cells (Wang et al., 2022; Singh et al., 2017). Since then, accumulating evidence has suggested that BNIP3L-mediated mitophagy played a momentous role in various other cell types developmental processes. However, the precise molecular mechanisms remained incompletely elucidated. Our knowledge about the function of mitophagy in DR is still in its nascent stages, yet it was evident that impaired mitophagy and inflammatory activation were observable in the diabetic patient retinas (Ashaari et al., 2018). In this study, we employed immunofluorescence and Western blotting techniques to demonstrate that downregulating SQSTM1 and upregulating BNIP3L expression significantly alleviated the progression of DR. These findings highlighted the potential of targeting the SQSTM1/BNIP3L axis as a therapeutic strategy against DR.

Molecular docking is an irreplaceable tool for assessing the binding interactions and affinities between a ligand and its target molecule. The computational analysis revealed that luteolin exhibited prominent binding activity with SQSTM1 and BNIP3L, which were promptly validated *in vivo* experiments. Luteolin is a flavonoid-based polyphenol recognized for antioxidant, antimicrobial, anti-inflammatory and anticancer properties. (Zhu et al., 2024; Imran et al., 2019; Mdsds et al., 2025; Caporali et al., 2022). These activities contributed to regulating various diseases, including cardiovascular disorders, neurodegenerative conditions and metabolic diseases (Dong et al., 2023). Currently, research on luteolin referred to as DR primarily focuses on its anti-inflammatory and antioxidant effects. In our current investigation, we observed that high-dose of luteolin demonstrated efficacy in reducing blood glucose levels and reversing weight loss, which were consistent with prior research (Zhang et al., 2024; Queiroz et al., 2021). Furthermore, luteolin proved effective in mitigating capillary permeability, improving retinal exudation, and hemorrhage and enhancing the BRB integrity in diabetic mice. Subsequent to these findings, our study unveiled that high-doses of luteolin led to a reduction in ROS levels and oxidative damage indicators in retinal tissues and serum assays. Accumulation of SQSTM1 has been associated with impaired autophagic flux, while reduced BNIP3L expression compromises mitophagy, exacerbating retinal oxidative stress and vascular dysfunction. By restoring the balance of this pathway, luteolin may mitigate BRB breakdown, reduce inflammatory responses, and attenuate the neurovascular damage characteristic of DR progression.

Although molecular docking results and expression analyses suggest that luteolin targets the SQSTM1/BNIP3L axis, definitive causal validation (e.g., via gene silencing or knockout approaches) is warranted. Future studies will focus on siRNA or CRISPR-Cas9-mediated manipulation of SQSTM1 and BNIP3L in retinal cells to confirm the necessity and specificity of this pathway in mediating the protective effects of luteolin. Although our study is based on *in vivo* validation, some mechanistic insights (such as those derived from molecular docking or literature-supported pathways) require further verification through targeted *in vitro* and clinical studies. In addition, the dosage, bioavailability, and long-term safety profile of luteolin remain to be fully established, particularly in the context of clinical application. Overall, this research lays a foundation for the development of innovative therapeutic strategies targeting the underlying molecular mechanisms of DR, with the potential to significantly improve outcomes for patients suffering from this sight-threatening complication of diabetes.

## 5 Conclusion

This study highlights the critical role of mitophagy and oxidative stress in the pathogenesis of DR, positioning luteolin, a natural flavonoid, as a potential therapeutic agent. Our findings demonstrate that luteolin modulates key protective pathways, notably the SQSTM1 and BNIP3L pathways, offering a novel approach to mitigating retinal damage. Through the integration of scRNA-Seq and comprehensive *in vitro* validation, we uncovered a deeper understanding of immune cell involvement, particularly monocyte-mediated mechanisms in DR progression. These insights expand the current knowledge of immune dysregulation in retinal pathology.

## Data Availability

The original contributions (scRNA-seq data) presented in the study are publicly available. These data could be downloaded from the NCBI repository, accession numbers GSE248284 and GSE245561.

## Abbreviations

AGEs, advanced glycation end products; AUC, area under the curve; ANOVA, a one-way analysis of variance; BRB, blood-retinal barrier; DR, diabetic retinopathy; DEGs, differentially expressed genes; EB, evans blue; ECs, endothelial cells; ELISA, enzyme-linked immunosorbent assay; ERG, electroretinography; GCL, ganglion cell layer; GO, gene ontology; H&E, hematoxylin and eosin; IF, immunofluorescence; IHC, immunohistochemistry; INL, inner nuclear layer; IPGTT, intraperitoneal glucose tolerance test; IPL, inner plexiform layer; KEGG, kyoto encyclopedia of genes and genomes; PAS, periodic acid-schiff; PBMCs, Peripheral blood mononuclear cells; PCA, principal component analysis; PKC, protein kinase C; PPI, protein-protein interaction; RBG, random blood glucose; RGCs, retinal ganglion cells; ROS, reactive oxygen species; scRNA-Seq, single-cell RNA sequencing; t-SNE, t-distributed stochastic neighbor embedding.
